# Persistence of bacterial indicators and zoonotic pathogens in contaminated cattle wastes

**DOI:** 10.1186/s12866-016-0705-8

**Published:** 2016-05-20

**Authors:** Giuseppe Blaiotta, Alessandro Di Cerbo, Nicoletta Murru, Raffaele Coppola, Maria Aponte

**Affiliations:** Dipartimento di Agraria, Università degli Studi di Napoli Federico II, Via Università 100, 80055 Portici (NA), Italy; Dipartimento di Chirurgia Generale e Specialità Chirurgiche, Università degli Studi di Modena e Reggio Emilia, Via del pozzo 71, 41124 Modena, Italy; Dipartimento di Medicina Veterinaria e Produzione Animali, Università degli Studi di Napoli Federico II, Via Foria 223, 80139 Naples, Italy; DAAA, Università degli Studi del Molise, Via de Sanctis, 86100 Campobasso, Italy

**Keywords:** Livestock wastes, Faecal indicators, Pathogens, *Mycobacterium avium* subsp. *paratuberculosis*, Storage, Environmental risks

## Abstract

**Background:**

Manure can provide a favourable environment for pathogens’ survival. De-contamination may be conducted by extended storage, until field conditions are suitable for application to land as source of agricultural nutrients.

**Results:**

The hygienic evaluation of manure and slurry coming from a plant that collects cattle livestock wastes from a big slaughterhouse was carried out. Samples were even collected from spillages in the area around the plant. Microbial analyses highlighted the massive presence of faecal indicators in all samples: mean counts of *Escherichia coli* and enterococci were always above EU limits for marketable processed manure products.

Cultures referable to the genus *Brucella* spp. were recorded in two samples of fresh manure but not in the aged ones. Conventional isolation techniques failed to detect members of the *Mycobacterium* genus, while by means of IS900 and F57 PCR real-time system on DNA directly extracted from environmental samples, the pathogen was detected in all cases.

**Conclusions:**

Thoughtful design of manure storage infrastructure is critical to prevent spills and over-topping of an open structure. The documented overload situation seems to lay the basis for an ongoing environmental contamination by enteric organisms and opportunistic pathogens circuit faecal-oral route. Moreover, the type of wastes analysed during this study, namely a mixture of fresh cattle manure, bedding and rumen content, needs a longer storage period or, alternatively, of specific chemical, biological or thermal treatments for stabilization.

**Electronic supplementary material:**

The online version of this article (doi:10.1186/s12866-016-0705-8) contains supplementary material, which is available to authorized users.

## Background

Outbreaks of food-borne diseases associated with the consumption of animal products have received much attention in North America and Europe [1]. Occurrences of human illness linked to *Salmonella* spp. in eggs, milk and meat products, to *Escherichia* (*E.*) *coli* O157:H7 in ground beef and to *Listeria* spp. in milk and soft cheeses, have prompted discussion on the adequacy of conventional methods of food inspection and the need for reducing food-borne pathogens in animal production systems to ensure food safety [1].

Historically, the focus of manure management has been on utilizing the nutrients in manure for crop production. Traditionally, manure is collected as slurry and is stored either in lagoons, above ground tanks, or earthen basins, until field conditions are suitable for its application to land as source of agricultural nutrients [1].

Manure from livestock and poultry contains a variety of pathogens; some are highly host-adapted and not pathogenic to humans, while others can produce infections in humans [2]. Pathogens can be transmitted to humans and animals directly by contact or indirectly by contamination of water or food. They can also be spread by the uncontrolled application of animal manure onto land, or during meat and milk processing. Contamination of the food supply may occur during slaughterhouse processing of infected animals [1].

The number and the type of microbial pathogens present in livestock wastes varies with animal species, geographic location of the farm, and the physicochemical composition of the manure. When animal manure and processing wastes are spread on land, microorganisms survival depends on manure type (solid or liquid), handling and treatment of manure, time of the year, presence or absence of plants, active microbial movements, microbial surface properties, soil water content, and environmental factors, such as soil pH, temperature and permeability [1]. As matter of fact, animal manure represents one of the major sources of water and air pollution. In particular, emission of greenhouse gases, leaching of nutrients and organic matter and pathogen contamination are the most important issues [3]. As the combined agricultural activity in the European Union (EU) produces more than 1.5 billion tonnes of animal manure every year, an effective solution is necessary for the disposal/treatment of manure [3].

Minimizing the potential for human illness from pathogens in manure requires understanding the survival characteristics of the various pathogens. Die-off of pathogens in manure and in the environment can range from days to years depending on the pathogen, the medium, and the environmental conditions [2].

*Mycobacterium* species are resistant to various physical conditions and are known for their ability to survive in the environment for a long time [4]. Members of the so called *Mycobacterium* (*M*.) *avium* complex are the causative agent of paratuberculosis, or Johne’s disease, in domestic and wild ruminants [5]. Johne’s disease is an economically important disease characterized by chronic intestinal inflammation, diarrhoea, progressive weight loss, emaciation, and death [6].

*M. avium* subsp. *paratuberculosis* (MAP) is consistently found in people with Crohn’s disease, suggesting that this agent is potentially zoonotic [4]. The main transmission route for ruminants is by ingestion of food or water contaminated with faeces from infected animals, including ingestion of MAP from contaminated pastures [6]. Once MAP is shed in faeces from the host, it is capable of surviving for prolonged periods in the environment [4, 7]. When the organisms reach the soil surface after slurry application, they interact in complex ways with the soil matrix [8, 9]. However, little is known about MAP survival after slurry application to soil. The first study under field conditions on the fate of MAP in agricultural soils after application of contaminated slurry [8] confirmed that the bacterium tends to remain on the grass and soil surface layers, moving very slowly through deeper soil layers. Moreover, according to findings, MAP is likely to move across the soil surface with rainfall runoff and to potentially contaminate surface water. Ground slope and soil type may influence the rate of MAP movement [8, 9].

In the United Kingdom, bedding and manure from premises under restriction should be sprayed with an approved disinfectant, then removed and stacked for at least three weeks prior to being spread. Ideally, slurry should be stored for a minimum of six months before being spread. Where possible, methods of spreading potentially infected manure and slurry should avoid airborne contamination (Eradication Programme for Bovine Tuberculosis - Commission Decision 2012/761/EU in accordance with Council Decision 2009/470/EC). In Italy, according to guidelines of the Ministry of Health, manure, slurry and bedding have to be stored at least five months before use as soil fertilizer (Ministerial Decree December 15 1995, no. 592; http://www.gazzettaufficiale.it/eli/id/1996/05/30/096G0314/sg).

The aim of the present survey was the hygienic evaluation of manure and slurry coming from a plant that collects cattle wastes as well as lumen contents coming from a plant, located in the South part of Italy, periodically deputed to the slaughtering of tuberculosis-infected cattle. Survival of faecal indicator bacteria and of three zoonotic pathogens was evaluated to provide new information *i)* on the safety of the long storage, as procedure for the production of aged manure and *ii)* on the reliability of the current indicators to monitor pathogens contamination in livestock wastes.

## Results and discussion

### Evaluation of microbial indicators of enteric contamination

Animal manure is widely applied to agricultural soil as a source of nutrients and organic matter. Inappropriate use of manure can lead to nitrate pollution of groundwater, eutrophication of surface waters, and transmission of pathogenic bacteria, viruses, or parasites to the soil environment [10]. Manure disposal certainly represents an issue to prevent outbreaks. De-contamination may be conducted either by the addition of proven disinfectants or, for very large quantities, extended storage for the long-term demise of pathogens. Thermal treatments constitute a more rigorous and reliable approach. Although less costly than originally expected, the use of such technology is still limited to specific areas of high risks [11]. As matter of fact, prolonged isolated storage for 4–6 months before land spreading is still the most common practice in Italy. This approach allows the number of pathogens possibly present in manure to decrease but not to totally disappear [11]. Moreover, after field application, manure-borne microorganisms can survive for two to three months at 5 to 25 °C [12]. Recently, the European regulation has been strengthened concerning the hygienic quality of recycled animal by-products like composted raw manure separated solids (Regulation No. 1774/2002 and modifications in Regulation No. 208/2006). According to European provisions, animal by-products must contain less than 5 × 10^3^*E. coli* or enterococci (*n* = 5, c = 1, m = 1000, M = 5000) per g of product and the absence of *Salmonella* in 25 g of product (*n* = 5, c = 0, m = 0, M = 0).

Where:

- *n* = number of samples to be tested;
- m = threshold value for the number of bacteria; the result is considered satisfactory if the number of bacteria in all samples does not exceed m;
- M = maximum value for the number of bacteria; the result is considered unsatisfactory if the number of bacteria in one or more samples is M or more; and
- c = number of samples the bacterial count of which may be between m and M, the sample still being considered acceptable if the bacterial count of the other samples is m or less.

**Fig. 1:**
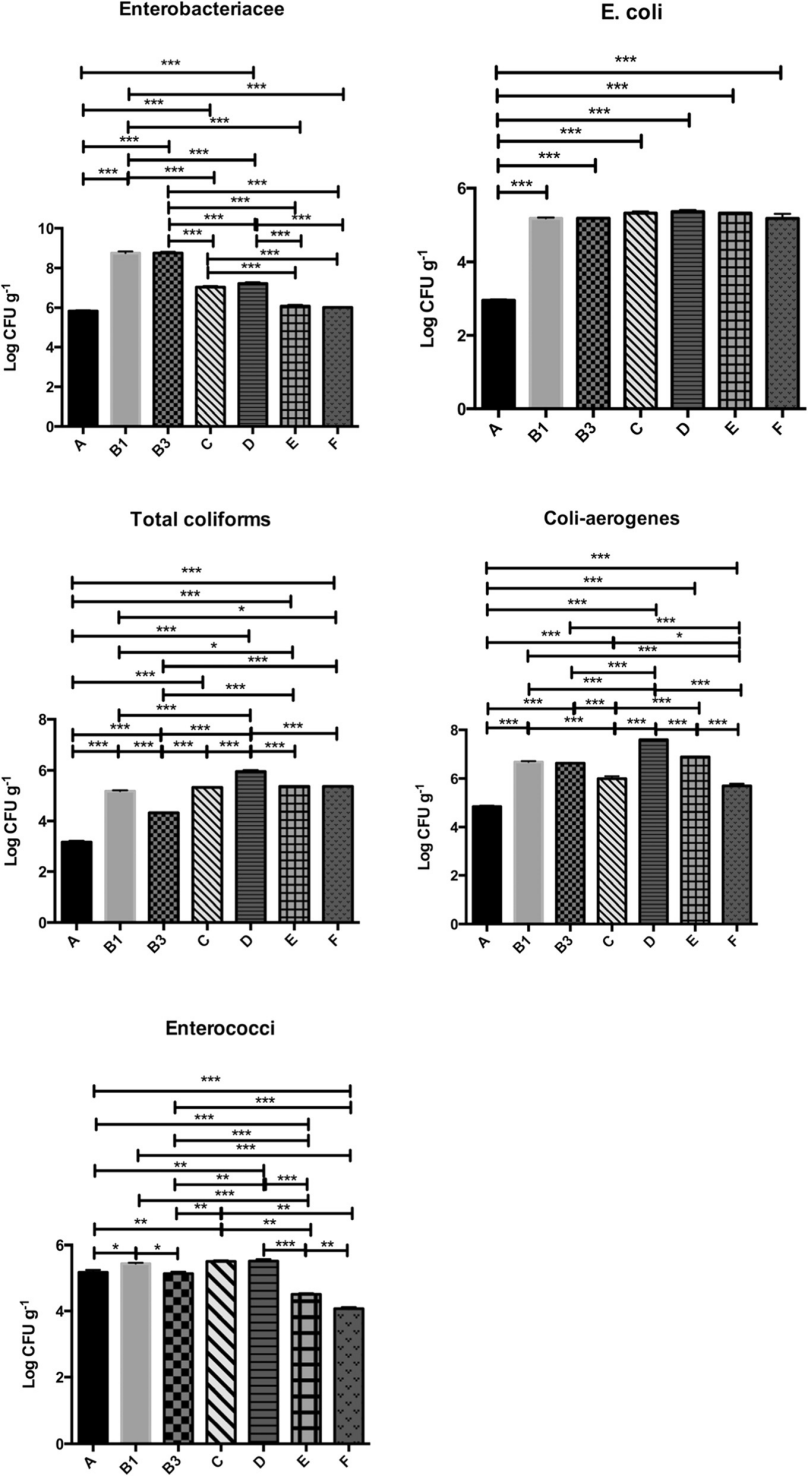
Schematic representation of bacterial indicators in the seven different sampling sites. *p < 0.05**; *p* < 0.01; ***p < 0.001

In the present study, CFU mean counts of *E. coli* and enterococci were in all cases above EU limits for marketable processed manure products (Fig. 1). In general, the concentration of enteric bacteria appears to be quite consistent in all samples (Fig. 1). Within the solid samples, the highest values were surprisingly recorded in samples coded as B1 and B3 and not in samples A collected near the dump site. Such unexpected outcomes may be likely due to the formation of a moisture-retaining crust, as well as, to the problematic draining of liquids, unavoidable in such kind of plan (Additional file 1: Figure S1). Moisture content of samples B1 and B3, collected downstream of A, was actually still high. (Additional file 2: Table S1). An initial increase phase (up to 1.5 orders of magnitude) in the microbial indicator counts in cattle manure has already been described. According to Sinton et al. [13], water content is the critical factor determining the growth period and magnitude, while temperature has a secondary role in determining the growth rate and duration. According to Wang et al. [14] the increases of magnitude for *E. coli* and faecal streptococci in dairy cow manure was of about 2.5 orders, but a survival-enhancing moisture level effect was demonstrated only for faecal streptococci. Freshly deposited faeces contain the nutrients required by enteric bacteria, so replication presumably depends on the cattle pat retaining water and attaining suitable temperatures for growth (the optimum temperature is around 35 °C for most enteric bacteria, although growth occurs at higher and lower temperatures). Thus, counts for fresh cow pats are likely to underestimate the loads on pastures during this period. When exposed to sunlight, the pats quickly formed a skin, which thickened to a well-defined crust within about 48 h. This crust helps to keep the interior of the pat moist and thus assists bacterial growth [13].

The low enterococcus counts, if compared to other faecal bacteria (Fig. 1), confirmed the limited value of these organisms as indicators in alternative to *E. coli*, as already reported by other authors [13]. In general terms, counts in solid samples (A, B1 and B3), except for enterococci and *Enterobacteriaceae* in samples B1 and B3 were always lower than those recorded in liquid sample E (Fig. 1). In the survey of McCarthy et al. [15], after separation of solid and liquid fractions from pig manure, *E. coli* and *Enterococcus* spp. counts were 10-fold lower in the manure solids than in the slurry, demonstrating that separation may be useful for reducing pathogen counts prior to composting. Remarkable (>10^4^ CFU ml^−1^) was the level of enteric contamination in samples collected out of the basins (C, D and F). Such outcomes come as no surprise since in several studies the transition of enteric pathogens from manure to soil [16] has been demonstrated. It should be noted that the CFU counts of *coli-aerogenes* group on VRBA were consistently higher than the values obtained for faecal coliforms by MPN method and reported as *E. coli* after confirmation by means of PCR (Fig. 1). According to the report of the Italian Istituto Superiore di Sanità [17], analytical techniques performed with liquid media, such as the MPN method, are, in such contexts, more reliable and repeatable than those using solid media, because of the consistency of the feature matrices analysed.

### Detection of *Salmonella* spp.

Presumptive *Salmonella* colonies were detected on both Salmonella Chromogenic agar and modified Brilliant Green Agar. After streaking on Kliger Iron Agar slant test, four different behaviours could be noticed (Table 1). A large majority of the cultures (17 out of 25) did not produced gas or H_2_S and exhibited a red slant combined with a yellow butt, thus indicating the fermentation of the sole glucose. According to key for identification, as supplied by Oxoid, such cultures were presumptively reported as *Shigella dysenteria* or *Shigella sonnei*. Six cultures out of 25 presented the same profile in the tube but combined with blackening due to H_2_S production. These isolates could be presumptively reported as *Salmonella typhi* or, alternatively, as *Proteus mirabilis*. The remaining two strains could be reported as *Citrobacter freundii* or, generically, as *Enterobacter* or *Salmonella* spp. To unequivocally assign cultures, a pool of selected strains was submitted to 16S rDNA sequencing (Table 1). With one exception, all sequenced strains presented a percent of similarity with known sequences higher than 98 %. According to results, strains presumptively reported as *Shigella* or *Morganella* or *Yersinia* spp. could be always reported to the species *Providencia stuartii*. Bacteria of this genus reside in soil, wastewater, and polluted water reservoirs; they have been also isolated from a broad range of living organisms. These opportunistic pathogens can cause acute enteric and urinary tract diseases, most often in young children and patients whose immune system was compromised by surgery or burns [18]. Four out of six strains, presumptively reported as *Salmonella typhi* or *Proteus mirabilis*, were sequenced. Two strains proved to belong to the species *Proteus mirabilis* and two to the genus *Providencia* spp., since presented the same similarity level with the following species: *Providencia vermicola* [NR.042415.1], *Providencia rettgeri* [NR.042413.1], *Providencia rustigianii* [NR.042411.1] or *Providencia stuartii* [NR.024848.1]. Indeed, the isolation of *Providencia* spp. from animal manure has already been reported [19], raising concerns about possible transmission of this pathogen to humans through food animals. In anaerobically digested sludge, *Salmonella* is usually retrieved in relatively low numbers (<10^3^ CFU g^−1^ dry weight) as compared to indicator bacteria such as faecal coliforms, faecal streptococci and enterococci (10^5^–10^6^ CFU g^−1^ dry weight) [20]. *Salmonella* inactivation rates are generally high during the various wastewater treatment processes [21]. *Salmonella* can survive up to three months in stored slurries, but the survival time does not encompass one month in biosolids applied to land [22]. In such optic, *Salmonella* does not appear as a good indicator. Indeed, available information on the correlation between the reduction of indicator bacteria and pathogens during biosolids treatment processes is often conflicting. Sorber and Moore [23] found higher inactivation rates of *Salmonella* in biosolids amended soil as compared to faecal indicators. On the other hand, Eamens et al. [24] reported that there was no correlation between *Salmonella* die-off and decline in *E. coli* or faecal streptococci numbers during the storage of biosolids or in biosolids amended soils and, even according to Sinton et al. [13], *S. enterica* is inactivated only slightly more rapidly than *E. coli* and other indicators. Likely, it would be better to use the decrease of indicator bacteria to monitor the efficiency of a treatment process, rather than the presence or absence of a pathogen, since there is very limited data on the existence or absence of correlation between pathogen die-off and the decline in bacterial indicator numbers in biosolids. Moreover, the lack of uniform pathogen assay techniques makes furthermore difficult to compare available information in the literature. As a matter of fact, in the present study, only the application of a genetic technique for identification - the 16S rDNA sequencing - allowed to report presumptive *Salmonella* colonies on selective media to different species within the *Enterobacteriaceae*.

**Table 1:** Results of tests on Kigler Iron agar for presumptive *Salmonella* cultures

N. isolate	Source	Reaction on Kigler Iron agar				Presumptive identification	Identification by 16S rDNA sequencing	% similarity
		Top	Bottom	Gas	H_2_S			
**1**	A	R	Y	–	*+*	*Salmonella typhi/Proteus mirabilis*	*Proteus mirabilis* NCTC 11938 (NR.043997.1)	99
2		R	Y	–	*–*	*Shigella dysenteria/Morganella* o *Yersinia* spp*.*		
3		R	Y	–	*–*	*Shigella dysenteria/Morganella* o *Yersinia* spp*.*		
4	B1	R	Y	–	*+*	*Salmonella typhi/Proteus mirabilis*		
**5**		R	Y	–	*–*	*Shigella dysenteria/Morganella* o *Yersinia* spp*.*	*Providencia stuartii* ATCC 29914 (NR.024848.1)	98
**6**		R	Y	–	*+*	*Salmonella typhi/Proteus mirabilis*	*Proteus mirabilis* NCTC 11938 (NR.043997.1)	85
21		R	Y	–	*+*	*Salmonella typhi/Proteus mirabilis*		
**7**	B3	R	Y	–	*–*	*Shigella dysenteria/Morganella* o *Yersinia* spp*.*	*Providencia stuartii* ATCC 29914 (NR.024848.1)	100
8		R	Y	–	*–*	*Shigella dysenteria/Morganella* o *Yersinia* spp*.*		
22		R	Y	–	*–*	*Shigella dysenteria/Morganella* o *Yersinia* spp*.*		
**9**	C	R	Y	–	*–*	*Shigella dysenteria/Morganella* o *Yersinia* spp*.*	*Providencia stuartii* ATCC 29914 (NR.024848.1)	99
10		R	Y	–	*–*	*Shigella dysenteria/Morganella* o *Yersinia* spp*.*		
11		R	Y	–	*–*	*Shigella dysenteria/Morganella* o *Yersinia* spp*.*		
23		R	Y	–	*–*	*Shigella dysenteria/Morganella* o *Yersinia* spp*.*		
12	D	R	Y	+	*+*	*Salmonella spp./Citrobacter freundii*		
**13**		R	Y	+	*–*	*Enterobacter* spp	*Raoultella terrigena* 84 (NR.037085.1)	98
**14**		R	Y	–	*+*	*Salmonella typhi/Proteus mirabilis*	*Providencia vermicola* OP1 (NR.042415.1); *Providencia rettgeri* DSM 4542 (NR.042413.1); *Providencia rustigianii* DSM 4541 (NR.042411.1); *Providencia stuartii* ATCC 29914 (NR.024848.1)	98
24		R	Y	–	*–*	*Shigella dysenteria/Morganella* o *Yersinia* spp*.*		
15	E	R	Y	–	*–*	*Shigella dysenteria/Morganella* o *Yersinia* spp*.*		
**16**		R	Y	–	*+*	*Salmonella typhi/Proteus mirabilis*	*Providencia vermicola* OP1 (NR.042415.1); *Providencia rettgeri* DSM 4542 (NR.042413.1); *Providencia rustigianii* DSM 4541 (NR.042411.1); *Providencia stuartii* ATCC 29914 (NR.024848.1)	99
17		R	Y	–	*–*	*Shigella dysenteria/Morganella* o *Yersinia* spp*.*		
25		R	Y	–	*–*	*Shigella dysenteria/Morganella* o *Yersinia* spp*.*		
**18**	F	R	Y	–	*–*	*Shigella dysenteria/Morganella* o *Yersinia* spp*.*	*Providencia stuartii* ATCC 29914 (NR.024848.1)	99
19		R	Y	–	*–*	*Shigella dysenteria/Morganella* o *Yersinia* spp*.*		
26		R	Y	–	*–*	*Shigella dysenteria/Morganella* o *Yersinia* spp*.*		

Strains in bold were submitted to 16S rDNA sequencing to confirm taxon

### Detection of *Brucella* spp.

After spreading on Brucella Selective Medium agar plates, presumptive *Brucella* spp. colonies could be retrieved in all samples (Data not shown). 16S rDNA sequencing confirmed the genus in three cases out of 21 (Table 2). Cultures referable to the genus *Brucella* spp. were retrieved in sample A and B1 but not in B3, which represent the manure ready to be used for agricultural purposes (Table 2).

**Table 2:** Results of identification by 16S rDNA sequencing of presumptive *Brucella* cultures

Number	Source	Closest relative	% similarity
A11	A	*B. microti* (NR.042549.1); *B. ce*ti (NR.042463.1); *B. pinnipedialis* (NR.042462.1); *B. suis* (NR.042461.1); *B. abortus* (NR.042460.1); *B. neotomae* (NR.043004.1); *B. melitensis* biovar *melitensis* (NR.043003.1); *B. canis* (NR.044652.1); *Och. intermedium* (NR.042447.1); *Och. cytisi* (NR.043184.1); *Och. lupini* (NR.042911.1); *Och. anthropi* (NR.026039.1); *B. ovis* (NR.036772.1); *Och. tritici* (NR.028902.1)	99
A11p		*Microbacterium xylanilyticum* (NR.042350.1)	97
A11g		*Microbacterium xylanilyticum* (NR.042350.1)	98
B11	B1	*Och. intermedium* (NR.042447.1); *Och. anthropi* (NR.026039.1); *B. microti* (NR.042549.1); *B. ce*ti (NR.042463.1); *B. pinnipedialis* (NR.042462.1); *B. suis* (NR.042461.1); *B. abortus* (NR.042460.1); *B. neotomae* (NR.043004.1); *B. melitensis* biovar *melitensis* (NR.043003.1); *B. canis* (NR.044652.1); *B. ovis* (NR.036772.1); *Och. tritici* (NR.028902.1)	99
B12		*B. microti* (NR.042549.1); *B. ceti* (NR.042463.1); *B. pinnipedialis* (NR.042462.1); *B. suis* (NR.042461.1); *B. abortus* (NR.042460.1); *B. neotomae* (NR.043004.1); *B. melitensis* biovar *melitensis* (NR.043003.1); *B. canis* (NR.044652.1); *B. ovis* (NR.036772.1); *Och. cytisi* (NR.043184.1); *Ochcpo lupini* (NR.042911.1)	99
B31	B3	*Providencia rustigianii* (NR.042411.1); *Providencia alcalifaciens* (NR.042053.1); *Providencia rettgeri* (NR.042413.1)	99
B32		*Providencia rustigianii* (NR.042411.1); *Providencia alcalifaciens* (NR.042053.1); *Providencia rettgeri* (NR.042413.1)	99
C11	C	*Providencia rustigianii* (NR.042411.1); *Providencia rettgeri* (NR.042413.1); *Providencia alcalifaciens* (NR.042053.1)	99
C12		*Providencia rettgeri* (NR.042413.1); *Providencia rustigianii* (NR.042411.1); *Providencia alcalifaciens* (NR.042053.1)	99
D11	D	*Providencia rettgeri* (NR.042413.1); *Providencia rustigianii* (NR.042411.1); *Providencia alcalifaciens* (NR.042053.1)	99
D12		*Providencia rustigianii* (NR.042411.1); *Providencia rettgeri* (NR.042413.1); *Providencia alcalifaciens* (NR.042053.1)	99
D13		*Providencia rettgeri* (NR.042413.1); *Providencia rustigianii* (NR.042411.1)	98
D14		*Myroides odoratus* (NR.044698.1)	96
E12	E	*Proteus myxofaciens* (NR.043999.1); *Proteus vulgaris* (NR.025336.1)	99
E13		*Proteus mirabilis* (NR.043997.1)	99
E14		*Providencia rettgeri* (NR.042413.1)	98
E15		*Proteus mirabilis* (NR.043997.1)	100
F11	F	*Proteus mirabilis* (NR.043997.1)	99
F12		*Proteus mirabilis* (NR.043997.1)	100
F13		*Proteus mirabilis* (NR.043997.1)	98
F15		*Providencia rustigianii* (NR.042411.1)	99

According to the Programme for the eradication, control and monitoring of Ovine and Caprine Brucellosis approved for 2013 by Commission Decision 2012/761/EU in accordance with Council Decision 2009/470/EC, in case of a positive result, measures to be taken in the farm include the collection and disinfection of the manure in a place far from the establishments. According to results, this practice seems to guarantee in samples of aged manure the absence of *Brucella* entities (Table 2). Two isolates from the shovelable basin could be reported to *Microbacterium xylanilyticum*, a species firstly isolated from a biofilm sample collected in a membrane bioreactor. Nine and six cultures were reported to the genus *Providencia* and *Proteus* spp., respectively. Both genera are not considered frank pathogens, unlike some of the other members of the *Enterobacteriaceae*, even though they are commonly isolated in clinical laboratories [24].

### Detection of *Mycobacterium* spp.

Members of the *Mycobacterium* genus grow slowly and compared with general bacteriological standards, require long incubation times, especially on primary isolation. In the present study, the Ziehl-Neelsen staining did not highlight the presence of *Mycobacteria* in the seven analyses samples (Data not shown). Actually, the increased risk of contamination from faster-growing species makes necessary to perform a decontamination of the sample or to use, as in this case, selective media with antibiotics, which has been proved to suppress the viability of not only contaminating species but also of *Mycobacteria* [3]. Using IS900 and F57 rt-PCR primers system for MAP identification, on DNA directly extracted from environmental samples, the pathogen was detected in all cases (Table 3). PCR, based on IS900, has been used for direct detection of MAP, without primary culture, from milk, faecal specimens, semen, and human intestinal tissue [25]. However, IS900-like genes have been found in other unrelated *Mycobacterium* species, thus proving that the PCR system used for IS900 is not fully specific for MAP [25]. To overcome this limit the set of IS900 primers was coupled with the one targeting the F57 gene and specific for MAP [25]. Anyway, the assay based on F57 gene proved to be less sensitive than that designed on IS900 (Table 3); this may be due to the different copy number of target sequences (15–20 copies for IS900 and only one for F57 gene) in the MAP genome [25]. In fact, the three PCR systems showed different sensitivity on pure DNA: 0.1, 0.3 and 1.0 MAP genomes for μl were detected for DHI, DH2 and DH3, respectively. Therefore, by considering the amount of analysed samples, the DNA isolation procedure, the DNA concentration used in the PCR reaction and the number of replicas for PCR reaction, the detection limits ranged from 10^3^ (DH1 and DH2) to 10^4^ (DH3) cells for gram or ml of sample.

**Table 3:** Results of real-time PCR (DHI, DH2 and DH3) for MAP detection

Sample	Primer set		
	DH1F-DH1R^a^ (IS900-69 bp)	DH2F-DH2R^a^ (IS900-65 bp)	DH3F-DH3R^a^ (F57 gene-80 bp)
A	+ + +^b^	+ + +	+ + −
B1	+ + +	+ + +	+ − −
B3	+ + +	+ + +	+ − −
C	+ + +	+ + +	- - -
D	+ + −	+ + +	- - -
E	+ + +	+ + +	+ + −
F	+ + +	+ + +	- - -

Tests performed in three independent PCR reactions on DNA extracted from seven samples (A, B1, B3, C, D, E, F) obtained by joining the corresponding five sampling units

^a^Primers described by Herthnek and Bölske (2006)

^b^PCR positivity (+) was confirmed by the sequencing of at least one PCR product per reaction replicates. Percent of similarity with reference MAP sequences in NCBI Genebank was at least 98 %

The positive finding of MAP DNA (Table 3) even in the aged manure (sample B3) could be explained not only by the ability of DNA to persist in the environment [26], but also by the daily incoming of fresh contaminated manure into the plant. Actually, exclusive findings of DNA without successful cultivation of viable cells can be, in the case of *Mycobacteria*, explained by several hypotheses. First, the detected DNA could be residual DNA released from dead cells, which does not represent any risk for the environment. Second, the DNA could originate from viable cells whose amount was under the limit of cultivability. Last but not least, ‘latent’ or ‘dormant’ phase of *Mycobacteria* infections represents a VBNC (Viable But Not Culturable) state in this pathogen [27]. From the data obtained, MAP was present in all analysed samples (Table 3), although according to the culture-based approach it was absent. Indeed, such inconsistencies are quite recurrent in the literature [3, 28]. Even if the sole presence of DNA may not have any effect on the safety use of the samples, it has to be considered the poor sensitivity of culture-based approaches in detecting low numbers of MAP cells. In such case, the use of slurry or manure for land fertilization or animal bedding could be hazardous [3]. Moreover, the potential risk to animals, and then to environment, cannot be excluded. Although transmission through inhalation respiratory is more common, the infection of cattle through consumption of herbage contaminated with their excreta has been proved [29] and so, the transmission by the oral route is believed to occur, by analogy, even in other species. For example, *M. bovis* infection is acquired by wild boar through feeding on pastures contaminated by cattle [30]. In other words, in addition to the direct risk to cattle, spreading of potentially infected manure or slurry on the land increases the risk of establishing a local wildlife reservoir of the pathogen, with consequent dangers of transmission to cattle. Already in 1933, Maddock [31] recovered infectious material even from faeces exposed to the elements for 178 days. Maddock concluded that faeces may be considered safe after about seven months of storage.

Farm yard (composted) manure need be exposed to a mean temperature of 60–70 °C for three weeks during composting to destroy *M. bovis* bacilli, and the majority of solid dung heaps does not reach this high temperature [32]. Thus composted manure cannot necessarily be considered safe and, according to EU Commission regulation EC 1774/2002 (2), heating to 70 °C for 60 min should precede anaerobic digestion to eliminate the risk of pathogens from spreading.

## Conclusions

The microbiological characteristics of the livestock wastes highlighted the massive presence of faecal indicators. Moreover, the detection of *Brucella* entities and of MAP by a culture independent PCR-based approach represents a serious concern. The overload situation, recorded and documented at the time of sampling seems to lay the basis for a continuing, albeit erratic, environmental contamination by enteric organisms and opportunistic pathogens circuit faecal-oral route. Likely, the type of wastes analysed during this study, namely a mixture of fresh cattle manure, bedding and rumen content, needs a longer storage period or, alternatively, of specific chemical, biological or thermal treatments for stabilization. Moreover, outcomes suggest that it is necessary to pay close attention to the type of bacterial indicator used to assess manure-associated risk, as well as to the analytical procedures adopted for the evaluation of the population level of bacterial indicators.

## Methods

### Description of the plant for slaughterhouse wastes management

The plant consists of two earthen concrete basins connected by a channel, positioned with the major axis running North-south. In detail, the first basin, sized about 15 × 6 m, is the point of collection of fresh cattle manure, bedding and rumen content (shovelled material). The discharge point is located on the North side. The opposite side (South) represents the site for the mechanical removal of the aged manure at the end of the cycle. On this side runs a concrete channel, about 4 m long, connecting the basin with a smaller one for the collection of slurry. This basin, sized about 4 × 3 m, is protected by a metal net. Both basins do not show waterproofing or rubber covers for the recovery of the biogas that, in these conditions, is slowly produced. Access to the manure disposal plan was allowed by the owner; a layout of the structure is provided in Additional file 1: Figure S1.

### Sampling

Seven locations were sampled in quintuple (*n* = 5) (see Additional file 1: Figure S1). The first three groups of solid samples (A, B1 and B3) were taken along the major axis of the basin harbouring the shovelable material. Sampling units, all coded as “A”, were collected in the North side of the basin, thus representing presumptively the material more recently spilled and characterized by a lower degree of drying. A second group of samples, “B1”, was collected in a middle position along the tub, thus it likely corresponded to a sample of material at an intermediate level of ripeness. The third sample, “B3”, was the manure as it appears at the time of recovery (about five months later). Two groups of liquid samples, “C” and “D” were collected by spillages in the area between the two basins. Samples “E” were collected from the basin for percolated slurries. Liquid samples, “F”, were taken from a spillover at valley of the plant.

The sampling was carried out under aseptic conditions with sterile equipment and containers. The picking of solid samples was performed approximately 10 cm below the surface. In detail, each manure sample unit consisted of 10 sub-units taken from different areas, at different depth, and thoroughly mixed. Slurry was collected by using a sampling probe. The process was repeated at least three times around the basin to create a composite sample unit in the vessel. Samples were then placed in sterile containers wrapped in tightly sealed plastic bag. The transport of specimens to the laboratory took place under refrigeration and protected from light to avoid changes of microflora due to regrowth phenomena. All samples were processed within 24 h.

### Microbial analyses

For each sample, the following parameters were evaluated: *Enterobacteriaceae*, total and faecal coliforms, *E. coli*, *Coli*-*aerogenes* group, faecal enterococci, and detection of *Salmonella*, *Brucella* and *Mycobacterium* spp. Each determination was performed on the five sampling units, except for *Salmonella*, *Brucella* and *Mycobacterium* spp., whose detections were carried on in triplicate, on specimens obtained by joining the five units collected for each sampling site. In detail, *Enterobacteriaceae* were evaluated by counting on Violet Red Bile Glucose Agar (VRBGA). Plates were incubated at 37 ± 1 °C for 24 h in microaerophilic conditions by overlay making with the same medium (ISO 21528–2:2004). Total coliforms were evaluated in Lactose Broth after incubation at 37 ± 1 °C for 24 + 24 h (ISO 9308–2). Tubes positive for gas formation were checked for faecal coliforms presence in Brilliant Green Bile Broth after incubation at 44 ± 1 °C for 24 + 24 h (ISO 4831:2006). To confirm the presence of *E. coli* in positive tubes, one loop of cells of each culture was subject to DNA extraction by boiling method according to protocol detailed by De Medici et al. [33]. Briefly, cells were resuspended in 300 μl of DNase-RNase-free distilled water (Sigma, Milan, Italy) by vortexing. The tube was centrifuged at 14,000 *g* for 5 min, and the supernatant was discarded carefully. The pellet was resuspended in 200 μl of DNase-RNase-free distilled water (Sigma) by vortexing. The microcentrifuge tube was incubated for 15 min at 100 °C and immediately chilled on ice. The tube was then centrifuged for 5 min at 14,000 *g* at 4 °C. The supernatant was transferred to a new microcentrifuge tube and incubated again for 10 min at 100 °C and chilled immediately on ice. An aliquot of 5 μl of the supernatant was used as the template DNA for the species-specific PCR assay as described by Kim et al. [34]. The level of faecal coliforms (*Coli*-*aerogenes* group) was confirmed by counting on Violet Red Bile Agar (VRBA) plates, after incubation at 32 ± 1 °C for 24 h in microaerophilic conditions (ISO 4832:2006). Enterococci were enumerated by using the selective medium Slanetz & Bartley agar after incubation at 37 ± 1 °C for 24 h (ISO 7899–2). All media and reagents were provided by Oxoid (Basingstoke, UK).

### Detection of *Salmonella* spp.

For the detection of *Salmonella* spp. the following procedure was adopted: pre-enrichment in Buffered Peptone Water (25 g o 25 ml of sample in 225 ml) with incubation at 37 ± 1 °C for 18 ± 2 h followed by enrichment in Muller-Kauffmann Tetrathionate broth (Oxoid). After incubation at 37 ± 1 °C for 24 h a loop of each culture was used to inoculate two selective media: Salmonella Chromogenic agar (Oxoid) and Modified Brilliant Green Agar (Oxoid). Plates were incubated at 37 ± 1 °C for 24 h (ISO 6579:2002 + A1:2007). Presumptive colonies were isolated, purified by repetitive streaking onto Salmonella Chromogenic agar plates and finally transferred in Kliger Iron Agar (Oxoid) slant test tubes. Results were collected after 24 h of incubation at 35 ± 1 °C. To confirm the presumptive identification, cultures were submitted to DNA extraction [33] and amplification of the 16S rDNA by Polymerase Chain Reaction (PCR). Conditions for amplifications were those described by Blaiotta et al. [35]. PCR amplicons were run on TBE agarose gels; plugs were excised from agarose, purified by QIAquick Gel extraction kit (Qiagen, Milan, Italy) according to the manufacturer’s instructions, and sequenced by Gene Chron (Roma, Italy). DNA similarity searches were performed with the National Centre of Biotechnology Information GenBank (http://blast.ncbi.nlm.nih.gov/Blast.cgi).

### Detection of *Brucella* spp.

For *Brucella* spp. detection, 20 g or 20 ml of solid (A, B1, B3) and liquid (C, D, E, F) samples, respectively, were homogenised in Tryptone Soya Broth (180 ml), and incubated for 24 h in microaerophilic conditions (5 % CO_2_). 10 μl of the enrichment broth were used to inoculate Brucella Selective Medium agar (Oxoid). Plates were incubated at 37 ± 1 °C for 24–48 h at 5 % CO_2_ [36]. Presumptive colonies were purified by streaking onto Tryptone Soya Agar (Oxoid) and confirmed by sequencing of the 16S rDNA. Procedures were the same previously described for *Salmonella* spp.

### Detection of *Mycobacterium* spp.

Solid (A, B1, B3) and liquid (C, D, E, F) samples were homogenised 1:10 in the enrichment medium Middlebrook 7H9 + ADC Middlebrook (Difco Laboratories, Detriot, MI) (180 ml) and incubated at 37 ± 1 °C under constant stirring for 15 days. Cultures were then streaked on Mycobacteria 7H11 agar plates containing OADC supplement (Difco). After incubation at 37 ± 1 °C for 10 days, typical colonies were transferred in 10 ml of Middlebrook 7H9 + ADC Middlebrook and further incubated at 37 ± 1 °C for 7 days [37]. At 4 and 7 days, one ml of culture was centrifuged and the cell pellet was analysed by Ziehl-Neelsen staining. MAP culture ATCC 19698 was used as positive control for the staining. Moreover, DNA was extracted from the seven samples, as obtained by joining the five sampling units, by means of FastDNA™ SPIN Kit for Soil (MP Biomedicals, Carlsbad, CA) and submitted to real-time PCR assays (DHI, DH2 and DH3) specific for MAP detection [25]. Real time PCRs were performed in a Chrom4 Real-Time PCR Detection System (Bio-Rad Laboratories, Milan, Italy) in triplicate. The reaction mixture included 100 ng of DNA template, 12.5 μl of iQTM SYBR Green Supermix (Bio-Rad), 0.75 μl of each primer (10 pmol/ml) and sterile water up to 25 μl. The cycling parameters were: 52 °C for 2 min, 95 °C for 10 min, followed by 45 cycles of amplification (95 °C for 15 s, 60 °C for 1 min). Fluorescence was read at the end of each amplification cycle. Melting curves were calculated from 65 to 94 °C, and read every 0.5 °C to check the amplified products. DNAs from MAP ATCC 19698 and *M. avium* subsp. *avium* ATCC 35718 were used as positive and negative control, respectively. Positive PCR samples (showing a melting curve similar to that of positive control) were separated by agarose (2 %) gel electrophoresis and products of the expected length were purified, sequenced and analysed as above described.

### Statistical methods

Data analysis was performed with GraphPad Prism version 6.0c (GraphPad Software, San Diego, CA, USA). A *p* < 0.05 was considered significant. All data, reported as Mean ± Standard Error of the Mean (SEM), were analysed by a one-way analysis of variance (ANOVA) with Bonferroni *post-hoc* correction.

## Additional files

Additional file 1: Figure S1. Schematic planimetry of the composting plan and sampling points. (DOCX 78 kb) Additional file 2: Table S1. Value recorded for pH and moisture content (%) in the seven analyzed samples. alues are means of three determinations (± sd) calculated with 95 % confidence. (DOCX 15 kb)

## Abbreviations

CFU: colony forming units
E: *Escherichia spp.*
M: *Mycobacterium spp.*
MAP: *M. avium* subsp. *paratuberculosis*
MPN: most probable number
PCR: polymerase chain reaction
rt-PCR: real time - polymerase chain reaction
VBNC: viable but not culturable
VRBA: violet red bile agar
VRBGA: violet red bile glucose agar
