# Supplementary figures and images for: Persistence of bacterial indicators and zoonotic pathogens in contaminated cattle wastes
Source: BMC Microbiol. 2016 May 20;16:87. doi: 10.1186/s12866-016-0705-8 (PMC4875618; doi:10.1186/s12866-016-0705-8)

**Supplementary figure 1**


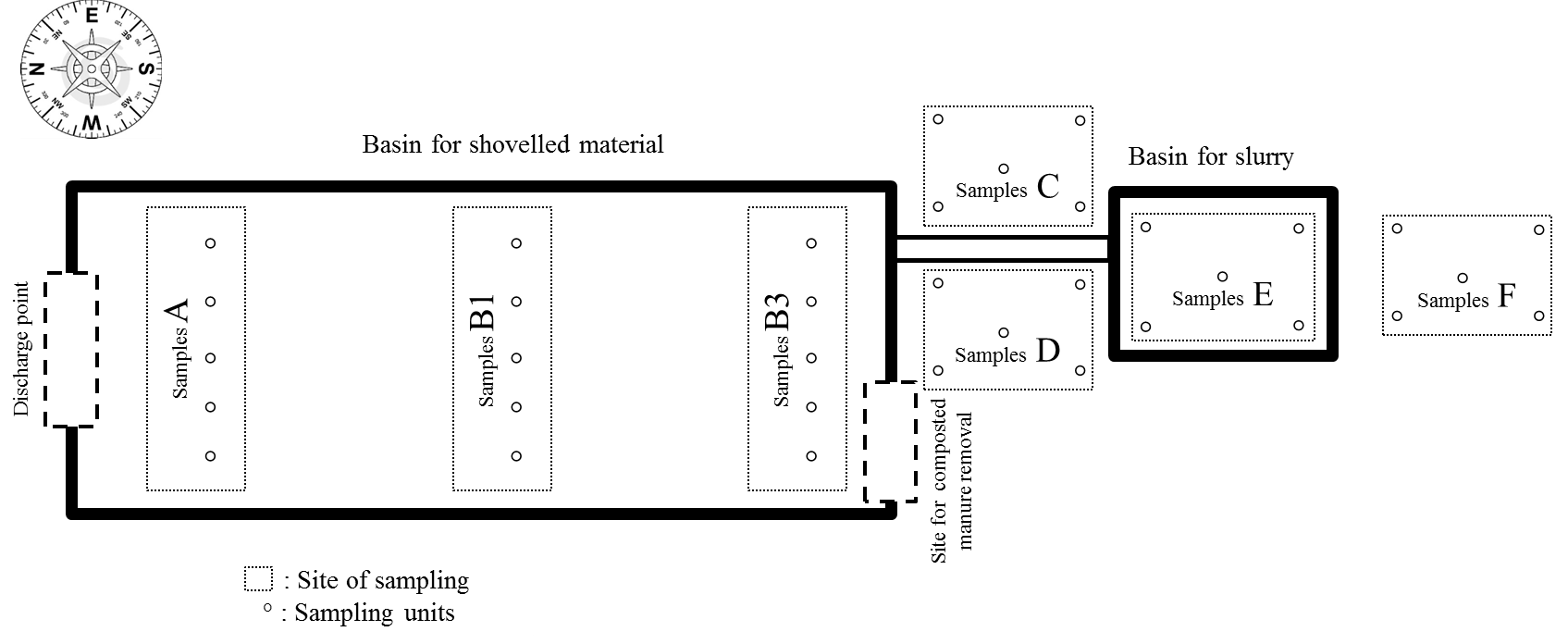

Supplement: Additional file 1: Figure S1. — Schematic planimetry of the composting plan and sampling points. (DOCX 78 kb) [file 12866_2016_705_MOESM1_ESM.docx]
