# Supplementary material for: Persistence of bacterial indicators and zoonotic pathogens in contaminated cattle wastes
Source: BMC Microbiol. 2016 May 20;16:87. doi: 10.1186/s12866-016-0705-8 (PMC4875618; doi:10.1186/s12866-016-0705-8)
Supplement: Additional file 2: Table S1. — Value recorded for pH and moisture content (%) in the seven analyzed samples. alues are means of three determinations (± sd) calculated with 95 % confidence. (DOCX 15 kb) [file 12866_2016_705_MOESM2_ESM.docx]

Suppl. Table 1. Value recorded for pH and moisture content (%) in the seven analyzed samples. alues are means of three determinations (± sd) calculated with 95% conﬁdence

| Code sample | Description | pH | Moisture content |
| --- | --- | --- | --- |
| A | Fresh manure | 7.4±0.1 | 85±2 |
| B1 | Intermediate manure | 8.0±0.3 | 86±3 |
| B3 | Aged manure | 8.4±0.2 | 86±1 |
| C | Liquid from puddle | 7.3±0.1 | 99±1 |
| D | Liquid from puddle | 7.6±0.1 | 99±0 |
| E | Liquid slurry | 7.1±0.0 | 99±0 |
| F | Liquid from puddle | 7.2±0.1 | 99±1 |
